# Development and tribological characterization of polyvinyl alcohol-based artificial cartilage for *in vitro* testing of viscosupplementation gels

**DOI:** 10.3389/fbioe.2026.1770162

**Published:** 2026-06-11

**Authors:** Marta Confalonieri, Lucrezia Bettella, Silvia Barbon, Elena Stocco, Silvia Spadoni, Daniele Pacchioni, Gabriele Vecchi, Andrea Porzionato, Silvia Todros, Piero G. Pavan

**Affiliations:** 1 Department of Industrial Engineering, University of Padova, Padova, Italy; 2 Department of Neurosciences, Section of Human Anatomy, University of Padova, Padova, Italy; 3 IBSA Farmaceutici Italia S.r.l., Lodi, Italy

**Keywords:** articular cartilage, friction, hyaluronic acid, polyvinyl alcohol hydrogel, viscosupplementation

## Abstract

**Introduction:**

Viscosupplementation is a treatment commonly used to restore joint lubrication in moderate osteoarthritis, although the effect of different intra-articular lubricants in friction reduction remains to be fully understood and tested *in vitro*. The aim of this study was to develop an artificial cartilage that mimicked the mechanical and tribological behavior of human articular cartilage, enabling *in vitro* evaluation of novel hyaluronic acid (HA)-based gels for viscosupplementation.

**Methods:**

Poly (vinyl alcohol) (PVA) hydrogels were evaluated by comparing different polymer concentrations (15%, 20%, and 25%) in water to identify which most closely replicates the compressive and frictional behavior of native articular cartilage.

**Results and Discussion:**

Scanning electron microscopy showed that higher polymer concentrations reduce pore size and increase surface irregularities, reflecting a denser network. Compressive testing indicated that the hydrogel stiffness rises progressively with polymer concentration, yielding compressive moduli within the physiological range of human cartilage. Tribological tests, performed using phosphate-buffered saline (PBS) as a lubricant under relevant loads and motion frequencies, revealed that friction also increases with polymer content. Among the tested concentration, 20% PVA hydrogel offers the most favorable combination of stiffness and lubricity, with a compressive modulus of approximately 300 kPa and a friction coefficient of about 0.14. Additional tests were performed by using four different HA-based viscosupplementation gels as lubricants. In all cases, a pronounced reduction in friction was observed, with coefficients decreasing to values between 0.02 and 0.06. In addition, microscopic surface analysis after testing showed a reduction in surface wear, confirming the protective and lubricating effect of HA-based gels. Overall, these findings indicated that PVA hydrogels, particularly at 20% concentration, can effectively be adopted as a reproducible and physiologically relevant equivalent of articular cartilage.

## 1 Introduction

Osteoarthritis is a progressive degenerative disease of the osteochondral tissue, affecting more than 7% of the global population in 2020 (Steinmetz et al., 2023). Since aging is the major osteoarthritis risk factor, the prevalence of this pathology is expected to rise sharply by 2050, with the incidence of knee osteoarthritis alone projected to increase by over 75% (Steinmetz et al., 2023). During the early stages, conservative management primarily aims to alleviate symptoms and maintain joint function through both non-pharmacological and pharmacological approaches. Non-pharmacological strategies typically include physiotherapy, regular physical activity, weight reduction, and the use of supportive orthotic devices, whereas pharmacological interventions often involve topical or oral non-steroidal anti-inflammatory drugs, intra-articular corticosteroid injections, and viscosupplementation with hyaluronic acid (HA) (Martel-Pelletier et al., 2016). These treatments are designed to reduce pain, enhance mobility, support patients’ daily activities and potentially postpone total knee arthroplasty.

Viscosupplementation has emerged as one of the most widely adopted intra-articular therapies for mild to moderate osteoarthritis (Leone et al., 2020). This procedure involves the injection of exogenous HA solutions into the affected joint to restore the viscoelastic properties of the synovial fluid, which decreases with the progression of the disease (Moreland, 2003). In osteoarthritic joints, both the molecular weight (Mw) and concentration of endogenous HA are substantially reduced, leading to compromised lubrication and limited shock absorption within the synovial cavity. Administration of exogenous HA not only replenishes these rheological deficiencies, but also stimulates the synthesis of endogenous HA and extracellular matrix (ECM) components, including proteoglycans and glycosaminoglycans (Moreland, 2003). To evaluate the potential of HA-based gels as effective joint lubricants, several aspects can be considered. Rheological properties, such as bulk viscosity and shear-thinning behavior, influence the lubrication mechanism and flow under stress (Bonnevie et al., 2019; Ren et al., 2022). Surface adhesive functionality affects chemical interactions at the interface, which can impact boundary lubrication and wear protection (Ren et al., 2022). However, Bonnevie et al. (2019) demonstrated that tribological testing can be more predictive for clinical purposes than experimental testing based on rheological or viscoelastic analyses, since friction tests can simulate physiological joint conditions and surface interactions. By characterizing the relationships among friction coefficient, time, sliding velocity, and applied load, a more comprehensive evaluation of lubrication performance can be provided. The tribological properties of HA formulations used for viscosupplementation have been extensively investigated using animal cartilage models (Mazurkiewicz et al., 2025; Mederake et al., 2022; Rebenda et al., 2020; Ren et al., 2022). However, such studies are constrained by several limitations, including high inter-sample variability, limited reproducibility, and the requirement for biosafety-compliant laboratory facilities (Jönsson et al., 2024; Pedersen et al., 2013). Furthermore, notable compositional and tribological differences exist between animal and human cartilage (Rajankunte Mahadeshwara et al., 2024). Consequently, there is an increasing interest in developing synthetic materials capable of replicating the mechanical and tribological behavior of human articular cartilage. Such materials would provide a more standardized and reproducible platform for evaluating the lubricating performance of HA-based and other viscosupplementation gels.

This study aimed at developing an artificial cartilage able to mimic the mechanical and tribological behavior of human articular cartilage for *in vitro* assessment of novel HA-based gels for viscosupplementation. For this purpose, poly (vinyl alcohol) (PVA) hydrogels were selected due to their suitable characteristics, including porous structure similar to articular cartilage, high water uptake, adjustable mechanical properties, low friction coefficient, and excellent biocompatibility (Grant et al., 2006; Wei and Dai, 2021). PVA hydrogels degrade very slowly *in vivo* and maintain long-term chemical stability in aqueous environments *in vitro* (Liang et al., 2024). Furthermore, different studies have reported that PVA mechanical behavior under compression, tension, and shear resembles the one of articular cartilage (Barbon et al., 2021; De Leon-Oliva et al., 2023; Spiller et al., 2011; Todros et al., 2022b).

The performance of PVA hydrogels as artificial cartilage can be optimized by adjusting several key parameters, including the polymer Mw, the degree of hydrolysis, and the overall concentration. Fully hydrolyzed PVA typically exhibits a hydrolysis degree of 98%–100%, whereas partially hydrolyzed PVA ranges from 87% to 89%. A higher degree of hydrolysis increases the density of hydroxyl groups available for hydrogen bonding, which enhances the stiffness of the hydrogel and stabilizes its network structure (Adelnia et al., 2022). Consequently, fully hydrolyzed PVA is preferentially used in cartilage engineering applications. PVA can also be classified based on its polymerization degree into four categories: ultrahigh (Mw 250÷300 kDa), high (Mw 170÷220 kDa), medium (Mw 120÷150 kDa), and low (Mw 25÷35 kDa) (Chen et al., 2021). Increasing the Mw enhances mechanical properties such as stiffness and strength, but reduces water solubility. Another critical aspect affecting PVA hydrogel mechanics is the polymer concentration in water (w/v), which in the literature ranges from 7% to 40%, being 15% the concentration most commonly adopted as artificial cartilage (Chen et al., 2021). However, the major limit of this concentration is the low value of compressive stiffness, which is generally below the one of articular cartilage, which ranges from 200 kPa to 5400 kPa (Belluzzi et al., 2023). This wide variability can be attributed to differences in mechanical testing conditions as well as to variations in anatomical site and tissue health status, distinguishing healthy from pathological cartilage. The instantaneous modulus of healthy cartilage can vary from 800÷1000 kPa in the trapezium of the metacarpal joint (Dourthe et al., 2019) up to 3500 kPa in the tibial plateau (Seidenstuecker et al., 2019), indicating that anatomical regions exposed to higher physiological loading typically exhibit increased compressive stiffness. Osteoarthritis consistently reduces compressive stiffness across all anatomical locations, with decreases of 40%–80% depending on disease severity (Belluzzi et al., 2023). For example, Ebrahimi et al. (2019) measured a compressive modulus of about 1200 kPa and 210 kPa, respectively for healthy and advanced osteoarthritic cartilage of the human tibial plate, thorough indentation tests.

In this work, starting from 15% PVA as reference material, we focused on PVA with medium to high Mw and increased the polymer concentration to obtain an acceptable value of the compressive stiffness, considering other two polymer concentrations (20% and 25%).

Considering tribological behavior, the friction coefficient of articular cartilage ranges from 0.005 to 0.025 for healthy joints, whereas values around 0.2 are considered representative of osteoarthritic conditions (Caligaris et al., 2009; Krakowski et al., 2024; Rajankunte Mahadeshwara et al., 2024). In a previous study, Li et al. (2010) investigated the frictional behavior of 15% PVA hydrogels, comparing it with human articular cartilage under cartilage-on-PVA, cartilage-on-cartilage, and cartilage-on-stainless steel contacts using a pin-on-plate experimental setup. They reported friction coefficients in the range of 0.076–0.178 for PVA hydrogels. However, literature data on the tribological behavior of PVA remain limited (Feng et al., 2022; Li et al., 2025; Luo et al., 2022).

In this study, PVA hydrogels with different polymer concentrations were prepared and physically crosslinked to develop an artificial cartilage. These hydrogels were then characterized with respect to their mechanical and tribological behavior, with the goal of identifying the concentration whose properties most closely mimic those of native articular cartilage. Once the compressive response and frictional performance of PVA hydrogels was thoroughly assessed, the optimal concentration was selected and its tribological behavior was then evaluated under lubrication with four (HA)-based gels. This approach enabled the development of a synthetic cartilage platform that can serve as a reliable basis for testing and evaluating viscosupplementation treatments.

## 2 Materials and methods

### 2.1 Sample fabrication

**FIGURE 1:**
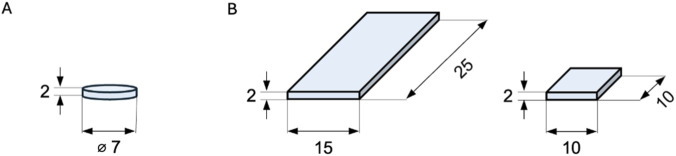
Shape and size (in mm) of samples for compressive **(A)** and friction **(B)** tests.

PVA powder (Mw 146,000–186,000 Da, 99+% hydrolyzed, Sigma Aldrich, St. Louis, MO, United States) was dissolved in deionized water for 8 h at 110 °C under stirring, ensuring complete dissolution. Solutions were prepared at three concentrations, 15%, 20%, and 25% (w/v). The PVA solutions were brought to 70 °C and then cast into 3D-printed polylactic acid molds to form 2 mm thick hydrogel membranes. Three freeze–thawing cycles were applied to induce hydrogel cross-linking, with each cycle consisting of a 24-h freeze at −20 °C and a 24-h thaw at 4 °C. The samples were subsequently stored at −20 °C until use. For mechanical testing, samples with different shape and size (Figure 1) were cut from the membranes; cylindrical samples were used for compressive tests, while rectangular and square samples were adopted for friction tests.

### 2.2 Scanning electron microscopy

The surface morphology of PVA hydrogels at concentrations of 15%, 20%, and 25% was investigated using scanning electron microscopy (SEM) before and after friction tests. The samples were fixed in 2.5% glutaraldehyde prepared in 0.2 M phosphate buffer (pH 7.4) for 24 h. The fixed hydrogels then underwent dehydration through a graded ethanol series ranging from 30% to 100%, followed by critical point drying. After preparation, the samples were examined by means of SEM (JSM-6490 L A, JEOL, Eching b. München, Germany) to assess their surface morphology.

### 2.3 Mechanical tests

All mechanical tests were performed using a Bose ElectroForce® Planar Biaxial Test Bench (TA Instruments, New Castle, United States).

#### 2.3.1 Compression and consolidation tests

**FIGURE 2:**
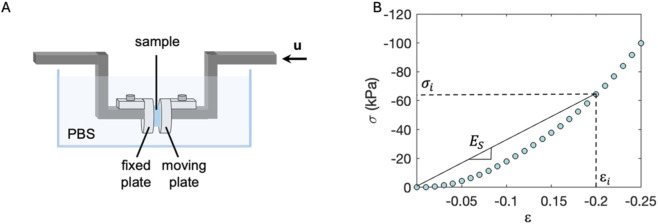
Schematic representation of the experimental setup used for compressive testing, with imposed displacement u **(A)**. The secant modulus (E_S_) was obtained for each sample as the ratio between the stress (σ_i_) and 20% strain (ε_i_), as illustrated on a representative stress–strain curve **(B)**.

The compressive tests were carried out on five samples for each PVA concentration. Cylindrical samples were allowed to thaw for 1 h immersed in phosphate buffer saline (PBS) and maintained in hydrated conditions throughout the whole duration of the test. One side of the sample was attached to a sample holder consisting of a flat fixed plate. Compressive tests were carried out by bringing a rigid flat moving plate into contact with the free surface of the sample (Figure 2A). The contact was assumed when a compressive force of 5 mN was detected, using a 22 N load cell. From this condition, the sample was compressed up to 20% strain at a strain rate of 150 %s^−1^, in order to evaluate the almost-instantaneous compressive response. The imposed strain was subsequently held constant for 500 s to evaluate relaxation phenomena. The maximum strain level was selected to mimic the physiological range corresponding to the maximum compressive strain for animal (Zevenbergen et al., 2018) and human (Kabir et al., 2021) cartilage. Force versus displacement data were acquired during the tests. Nominal stress σ was calculated as the current force divided by the nominal transversal area of the sample, while nominal strain (ε) was determined as the displacement divided by the initial sample height. The median nominal stress and the corresponding interquartile range (IQR) were calculated for each PVA hydrogel concentration. For the comparison of PVA stiffness, the median secant modulus (E_S_) (IQR) was calculated for each sample as the ratio of the stress to the strain at 20% of strain (Figure 2B). In the consolidation data analysis, the median compressive force and the associated IQR was calculated for each PVA hydrogel concentration.

#### 2.3.2 Friction test

**FIGURE 3:**
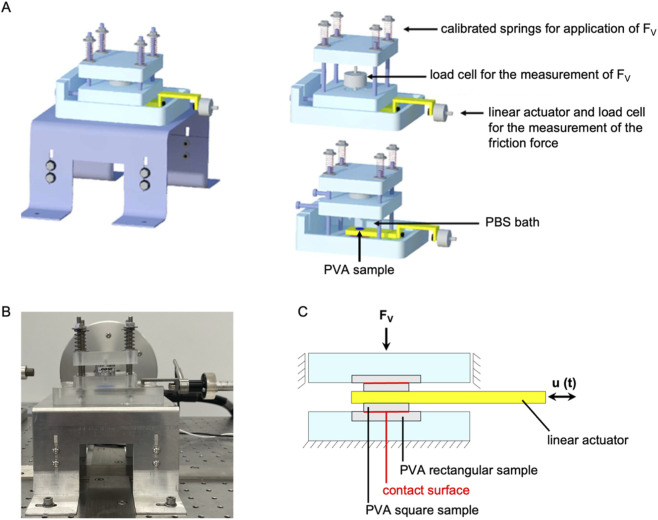
Custom-made experimental setup for friction testing. **(A)** General view and detailed 3D representation of the setup. **(B)** Image of the assembled setup. **(C)** Schematic representation of the test configuration: two PVA square samples were attached to the linear actuator, while two rectangular samples were fixed to the supports. The regions of contact between the coupled PVA surfaces were indicated in red. A vertical compressive force F_V_ was transmitted normally to the PVA contact surfaces and a displacement *u*(t) was applied by the linear actuator during testing.

The tests were performed using a custom-designed experimental set-up (Figure 3) integrated with the Bose ElectroForce® Planar Biaxial test bench. The system was designed to apply a vertical compressive force that induced the contact between coupled PVA surfaces during their relative motion in the tangential direction (horizontal). The coupling occurred between a rectangular sample fixed in the horizontal direction and a square sample driven horizontally by a linear actuator. The setup allowed for two simultaneous couplings, located at the lower and upper parts of the actuator (Figure 3C). The vertical compressive force was generated by four calibrated springs adjusted through micrometric screws and continuously measured by a load cell with a full-scale of 400 N. During the test, the samples were fully immersed in a PBS bath. For each experiment, the displacement *u*(t) was imposed by the linear actuator, while the resulting friction force was recorded by a load cell with a full-scale of 22 N.

**FIGURE 4:**
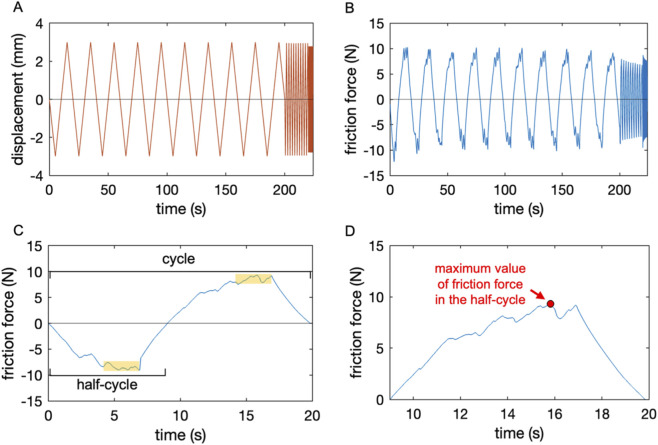
Representative analysis of friction test data. Raw experimental measurements showing **(A)** friction force vs*.* time and **(B)** displacement vs*.* time for all three tested frequencies (low: 0–200 s; medium: 200–220 s; high: 220–224 s). **(C)** Friction force vs*.* time for a single cycle, with the yellow area indicating the time range where the force was almost constant. **(D)** Friction force vs*.* time for a single half-cycle, with the red dot indicating the maximum friction force within the interval.

Friction experiments were performed on PVA hydrogels with concentrations of 15%, 20%, and 25%. For each PVA concentration, the tests were carried out on five samples, each comprising two rectangular and two square membranes (Figure 1B). The samples were thawed for 1 h by immersion in PBS at RT and then maintained hydrated. Each specimen was fixed onto the experimental system using cyanoacrylate adhesive to ensure stable positioning. Each sample was then allowed to equilibrate within the experimental setup for 180 s prior to testing in order to standardize the loading conditions. Tests to evaluate the PVA friction coefficient depending on polymer concentration were carried out in PBS. Furthermore, friction tests were developed also using four specific HA-based gels (IBSA Farmaceutici Italia S. r.l, Lodi, Italy) as lubricants, labelled as HA1, HA2, HA3, and HA4 in the following. In total, 15 material-lubricant combinations were considered. In all the experiments, calibrated springs were deformed to achieve a vertical compressive force of 20 N and a cyclic displacement with a nominal range of 6 mm (−3 mm to +3 mm) was applied by the linear actuator. The displacement cycles were applied sequentially at three frequencies: 0.05 Hz, 0.5 Hz, and 2.5 Hz (hereon indicated as low, medium and high frequency, respectively), with 10 cycles in each condition (Figure 4A). The frequencies were selected based on previously published tribological procedures and reproduced physiologically relevant loading conditions (Balazs, 1974; Finelli et al., 2011).

The acquired data, recorded in terms of friction force over time (Figure 4B), were analyzed through a user-defined script in MATLAB (MathWorks, Natick, MA, United States) to calculate the friction coefficient for each material-lubricant combination. Starting from rough data of friction force versus time in all the cycles, the script was conceived to extract a single measure of friction force from each half-cycle. Indeed, during a whole cycle, the displacement imposed by the linear actuator was inverted and the friction force was subjected to fluctuations, showing a more stable behavior in between the limits of the displacement range, as shown in Figure 4C. In order not to underestimate the friction force, its maximum value was chosen within each half-cycle (Figure 4D), while the vertical compressive force of each half-cycle was obtained as a linear interpolation of the data recorded over time, which showed a slight decrease of vertical compressive force due to creep phenomena in PVA hydrogels.

The friction coefficient of each half-cycle was then computed as half of the ratio between the friction force and the vertical compressive force, in order to account for the two simultaneous couplings. Finally, a mean value of the friction coefficient was calculated over the 20 half-cycles at each frequency. For each PVA hydrogel concentration and for each frequency, the median friction coefficient among the five samples and the corresponding IQR were calculated.

### 2.4 Statistical analysis

Because of the limited number of tested samples, statistical comparisons were performed using a non-parametric one-way ANOVA (Kruskal–Wallis test) followed by *post hoc* analyses using the Dunn test, with an experiment-wise significance threshold set at 0.05 to identify differences among the experimental groups.

## 3 Results

### 3.1 PVA hydrogels morphology

**FIGURE 5:**
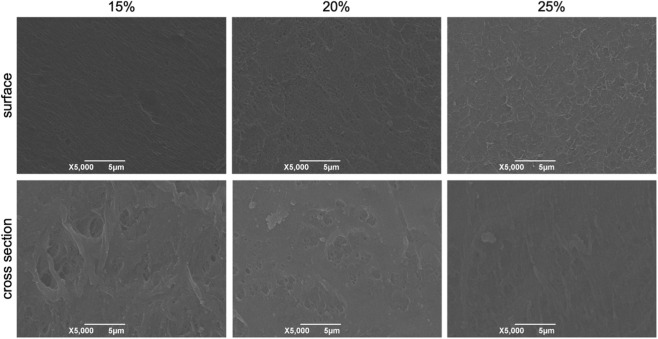
SEM analysis of the superficial and cross-sectional morphology of PVA hydrogels at 15%, 20% and 25% concentration. Scale bars: 5 μm.

The surface of cross-linked PVA hydrogels at 15%, 20%, and 25% concentration was investigated using SEM, qualitatively highlighting a morphology variation at microscale across all samples (Figure 5). Surface morphological irregularity increased with the PVA concentration. Cross-sectional analysis suggested a low porosity with decrease in pore size and a progressively more compact internal structure as the polymer concentration was increased.

### 3.2 PVA compressive behavior

**FIGURE 6:**
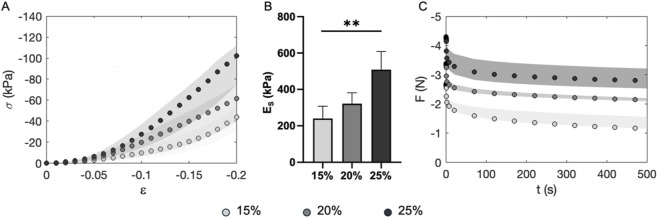
Results of compression and consolidation tests carried out on PVA hydrogels at 15%, 20%, 25% concentration (n = 5 per group). **(A)** Median instantaneous nominal stress (σ) ± interquartile range (IQR) vs*.* nominal strain (ε). **(B)** E_S_ calculated at 20% strain (median ± IQR). **(C)** Median compressive force ± IQR vs*.* consolidation time. **p < 0.01.

The almost-instantaneous compressive behavior of PVA hydrogels at varying polymer concentrations is shown in Figure 6A. Hydrogel stiffness, estimated using the E_S_, at a compressive strain of 20%, was 240 (172–308) kPa, 322 (262–382) kPa, and 509 (409–609) kPa for 15%, 20%, and 25% PVA, respectively (Figure 6B). A significant difference was detected between PVA at 15% and 25% (p < 0.01). These results suggested a trend of increasing stiffness with higher PVA concentrations. The compressive force versus time for 15%, 20%, and 25% PVA is shown in Figure 6C. In particular, the times of 180 s and 400 s correspond to the initial and final instants of friction tests. Whitin this range, the measured force decreased by 5%, 3%, and 1% for PVA hydrogels at 15%, 20%, and 25% concentrations, respectively.

### 3.3 PVA frictional behavior

**FIGURE 7:**
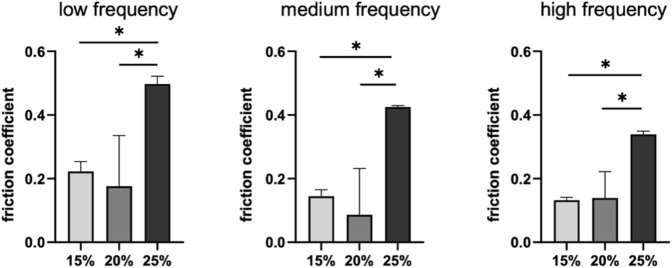
Comparison of the friction coefficient of PVA hydrogels with different concentrations (15%, 20% and 25%): median friction coefficients ± IQR tested under low (0.05 Hz), medium (0.5 Hz) and high (2.5 Hz) frequencies (n = 5 per group). PBS was used as lubricant in all experiments. *p < 0.05.

Friction tests of PVA hydrogels with different concentrations (Figure 7) showed that, across the three different tested frequencies, the friction coefficient exhibited an increase with higher PVA concentrations. For example, at low frequency, the PVA friction coefficient in PBS was 0.223 (0.216–0.231), 0.176 (0.175–0.333), and 0.497 (0.491–0.521) for 15%, 20%, and 25% PVA, respectively. All the obtained values of PVA friction coefficient also at medium and high frequencies are provided in the Supplementary Material. At high frequency, the displacement reached approximately ±2.8 mm possibly due to inertial effects of the system. However, this did not affect the measured friction forces which were evaluated over a subinterval of relative displacement that always remained between 0 mm and ±2.8 mm for all the frequencies. *Post-hoc* analysis revealed statistically significant differences between the friction coefficients of PVA hydrogels at 15% and 20% concentration, compared with 25% concentration (p < 0.05), for all the tested frequencies.

**FIGURE 8:**
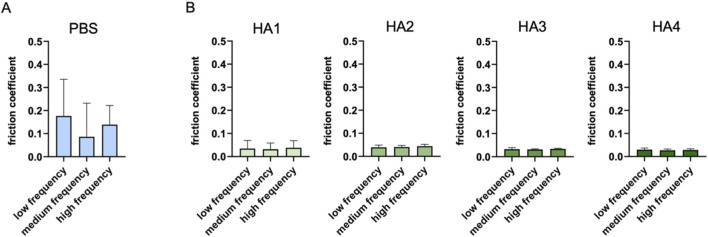
Comparison of the median friction coefficient ± IQR of PVA hydrogel at 20% concentration measured at three frequencies: low (0.05 Hz), medium (0.5 Hz), and high (2.5 Hz) (n = 5 per group). The lubrication is obtained with PBS **(A)** or with HA-based gels (HA1, HA2, HA3, and HA4) **(B)**.

The influence of testing frequency on the tribological behavior of PVA hydrogels was further examined. Results for PVA at 20% concentration are presented here, whereas data for the 15% and 25% concentrations are provided in the Supplementary Material. A frequency-dependent variation in the friction coefficient was detected when PBS was employed as a lubricant, suggesting a higher friction coefficient at lower frequency (Figure 8A). The friction coefficient of PVA at 20% concentration in PBS was 0.176 (0.175–0.333), 0.086 (0.085–0.201), and 0.139 (0.099–0.192) at low, medium and high frequency, respectively. However, due to a large variance of the data, this difference was not statically significant in the case of PVA at 20% concentration. In contrast, the use of HA-based gels resulted in negligible changes in the friction coefficient across the tested frequencies (Figure 8B).

**FIGURE 9:**
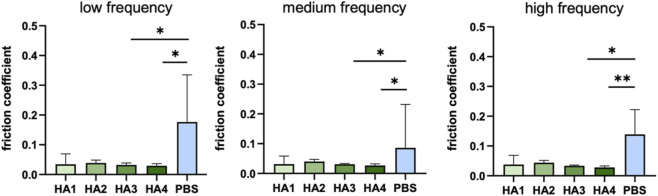
Comparison of median friction coefficient ± IQR of PVA hydrogel at 20% concentration under different lubrication conditions, i.e., PBS or HA-based gels (HA1, HA2, HA3, HA4) (n = 5 per group). Tests were carried out at low (0.05 Hz), medium (0.5 Hz), and high (2.5 Hz) frequencies. *p < 0.05, **p < 0.01.

Moreover, for each testing frequency, the effect of the lubricant on the PVA friction coefficient was evaluated. The results showed that the use of HA-based gels decreases the friction coefficient in comparison with PBS (Figure 9). Statistically significant differences were observed only between HA3 and HA4 gels and PBS (p < 0.05). However, considering the trends among groups, the absence of wider statistical significance likely reflected the reduced sample size rather than the absence of an actual effect.

### 3.4 PVA hydrogels wear

**FIGURE 10:**
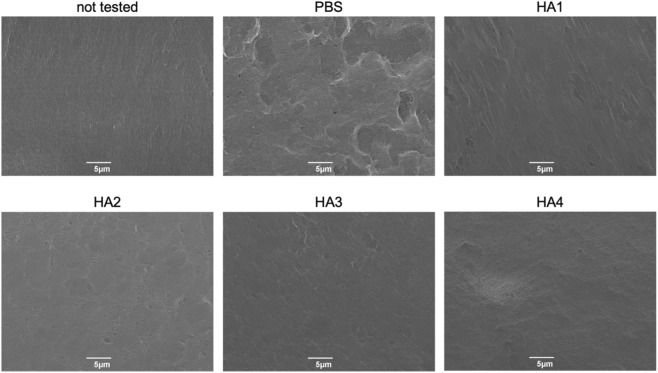
SEM images of the surface of PVA hydrogels at 20% concentration before and after friction test, carried out in different lubrification conditions: PBS, HA1, HA2, HA3, HA4. Scale bars: 5 μm.

The use of HA-based gels as lubricants reduced the friction coefficient and it was thus expected to limit PVA surface wear. To assess this effect, the surfaces of PVA hydrogel samples were observed with SEM after being subject to friction tests in different lubrication conditions (PBS, HA1, HA2, HA3, and HA4). The surface morphology of PVA hydrogels at 20% concentration was compared before and after testing under lubrication with PBS or HA-based gels (Figure 10). All samples exhibited surface morphological changes after friction tests to an extent depending on the lubricant adopted. Friction measurements in Section 3.3 revealed that the use of HA-based gels reduces the friction coefficient by approximately one order of magnitude, compared to PBS. Post-test SEM images were consistent with these findings: surfaces tested in PBS appeared more irregular and worn than those lubricated with HA. Among the different HA-based gels, the superficial damage was similar.

## 4 Discussion

PVA hydrogels with different concentrations were characterized to identify the best compromise in terms of compressive stiffness and tribological behavior.

Microscopic analysis of cross-linked PVA hydrogels revealed a microstructured porous surface, with surface irregularities increasing with the polymer concentration. Cross-sectional images indicated a gradual reduction in the pore size and a more compact internal architecture at higher concentrations. These trends are consistent with previous studies, which reported that the increasing PVA concentration enhances network crosslinking and crystallinity, leading to denser polymer meshes with smaller pores (Duque-Ossa et al., 2021; Li et al., 2017; Wan et al., 2014). Surface roughness, qualitatively assessed on PVA surface, influences the effective contact area during surface sliding and contributes to the entrapment and retention of lubricating fluid within the surface irregularities, thereby contributing to sustain lubrication. Differently, bulk porosity, assessed in cross-sectional images, allows for fluid uptake and redistribution within the hydrogel matrix, leading to swelling effects that can influence the compressive behavior.

The mechanical of PVA hydrogels is intrinsically related to their microscopic architecture; as the polymer network becomes more compact, the resulting material typically exhibits greater stiffness (Wan et al., 2014). Mechanical tests carried out to assess the compressive behavior of PVA hydrogels as a function of their concentration showed that the PVA hydrogel stiffness, expressed by E_S_ at a compressive strain of 20%, increased from 270 kPa to about 510 kPa, with growing PVA concentration. These values are within experimental data reported in the literature for the compressive stiffness of human articular cartilage, which cover a wide range, from approximately 200 kPa–5400 kPa (Belluzzi et al., 2023); this is mostly due to differences in testing methodologies, tissue composition, and inherent variability of biological samples. Several studies on healthy knee articular cartilage report compressive stiffness values of about 500 kPa and 800 kPa, measured through unconfined and confined compression tests, respectively (Jurvelin et al., 2003; Nissi et al., 2007; Zimmerman et al., 2021). Therefore, among the investigated PVA hydrogels, those with the higher polymer concentrations, demonstrating higher stiffness, can be considered the best solution to mimic the physiological properties. However, these values still fall toward the lower end of the range reported for native cartilage.

Friction measurements were carried out on PVA hydrogels at concentrations of 15%, 20%, and 25%, using PBS as a lubricant. While 15% and 20% PVA hydrogels showed similar friction coefficients at all test frequencies, 25% PVA exhibited a significantly higher friction coefficient. This effect can be due to the higher polymer concentration, which increases the crosslink density (Gong, 2006), but also prevents the solubilization of the polymer in water during the preparation process, thus inducing higher inhomogeneity and surface irregularities. Indeed, polymer concentration in water is a critical factor influencing the mechanical behavior of PVA hydrogels. The dissolution of PVA for hydrogel preparation becomes increasingly challenging at higher concentrations: solutions with lower polymer content exhibit reduced viscosity, facilitating processing but producing hydrogels with comparatively weaker mechanical properties (Goldberg et al., 2017). Above approximately 15%, PVA powders or granules tend to aggregate, forming lumps that hinder dissolution, and at concentrations of 30÷40% effective processing and molding become very difficult (Adelnia et al., 2022). In addition to polymer content, water plays a major role in determining both the macroscopic mechanical properties and the microscopic dynamics of PVA chains. For instance, Li et al. (2021) reported that even 1.8% water can decrease the tensile strength of PVA by roughly 32%.

Friction coefficients measured for the 15% and 20% PVA samples in PBS are in agreement with previously reported values for PVA in contact with articular cartilage by Li et al. (2016), Li et al. (2010). In these works, they investigated the tribological behavior of 15% PVA hydrogels in comparison with human articular cartilage using a pin-on-plate setup under cartilage-on-PVA, cartilage-on-cartilage, and cartilage-on-stainless steel contacts, reporting friction coefficients in the range of 0.076÷0.178, comparable with the friction coefficient range obtained in the present study.

In the literature, a wide variety of experimental configurations are employed for friction testing, including pin-on-disk and ball-on-disk systems. While these configurations differ in geometry, most involve cyclic motion (Li et al., 2016; Li et al., 2010; Li et al., 2021; Mamada et al., 2011), comparable to the experimental setup used in the present study. Experimental parameters are typically reported in terms of pin geometry, sliding speed, and total test duration, whereas the sliding distance per cycle is less frequently indicated. The sliding distance is similar to the one adopted in our study. In particular, our sliding distance per cycle (6 mm) falls well within the range of values reported in similar studies, such as Li et al. (2010), adopting a 4 mm sliding, and Li et al. (2024), adopting a 10 mm sliding.

Physically cross-linked PVA hydrogels typically exhibit a viscoelastic behavior (Todros et al., 2022b; 2022a). To evaluate possible time-dependent effects on the measured tribological properties, preliminary experiments were performed to characterize stress relaxation phenomena in a time range including friction test interval. The limited amount of compressive force decrease in this time range indicates that friction data are not affected by viscoelastic phenomena. Furthermore, the normal force was continuously recorded throughout the experiment, and the friction coefficients were calculated as the ratio of the friction force to the corresponding instantaneous normal force. This approach ensured that any fluctuations in normal loading conditions during the experiments were inherently incorporated into the data processing and evaluation.

Therefore, 20% PVA hydrogel exhibited compressive stiffness within the physiological range of articular cartilage, along with a relatively low friction coefficient approaching that reported for osteoarthritic cartilage (Caligaris et al., 2009). These results indicate that PVA at this concentration is a promising candidate for replicating the tribological behavior of articular cartilage. In particular, PVA hydrogels are better suited as a model of pathological tissue, exhibiting both compressive and tribological characteristics comparable to those of osteoarthritic cartilage (Belluzzi et al., 2023; Caligaris et al., 2009) and can thus serve as a platform for testing how different lubricants can reduce the friction coefficient toward values typical of normal cartilage. Based on these considerations, 20% PVA hydrogel was selected as the reference material to assess the lubricating performance of four HA-based gels for viscosupplementation treatment.

While the friction coefficient of 20% PVA hydrogel in PBS was varying with test frequency, the use of HA-based gels resulted in similar values of friction coefficient across the tested frequencies, consistently with the literature (Rebenda et al., 2020).

Friction tests carried out to compare the effect of different lubricants revealed that the use of HA-based gels markedly decreases the friction coefficient of PVA hydrogels, showing median values that range from 0.027 to 0.065. These friction coefficients are closer to physiological levels reported for healthy cartilage, which range between 0.002 and 0.01 (Oungoulian et al., 2015), than the friction coefficients of PVA hydrogels in PBS. These results are also consistent with studies showing that HA can reduce friction at both cartilage-on-cartilage (Bell et al., 2006) and hydrogel-on-cartilage interfaces (Li et al., 2016) compared to saline solutions. In the present study, the differences between PBS and HA were evident; despite this, not all comparisons reached statistical significance, likely due to the limited sample size and the large number of groups included in the *post hoc* analyses. Future studies will expand the sample cohort to strengthen these observations.

The wear of PVA hydrogels after friction tests was evaluated by means of SEM to assess surface differences depending on the lubricant used. Wear in PVA hydrogels occurred as visible surface degradation resulting from the relative motion between contact surfaces. SEM analysis after friction tests showed that the surfaces of samples tested in PBS exhibited greater degradation and more irregularities compared to those lubricated with HA. These results confirm the friction reduction obtained with HA lubricants with respect to PBS. The observed wear patterns under PBS lubrication are consistent with previously reported findings under similar experimental protocols (Li et al., 2016; Sardinha et al., 2013). Among the different HA-based gels, the extent of superficial damage was qualitatively similar across all groups.

Despite these promising results, this study presents some limitations that will be addressed in future developments. First, the mechanical and tribological properties of PVA hydrogels can be further optimized, by incorporating chemical cross-linkers or applying thermal treatments to enhance the hydrogel stiffness, and by modifying the PVA surface to reduce the friction coefficient.

Moreover, the vertical compressive force applied during friction tests was relatively low, being nonetheless similar to the normal load applied in the literature to assess the tribological behavior of biomaterials for cartilage replacement (Li et al., 2017; Li et al., 2010). However, articular cartilage experiences considerably higher contact stresses *in vivo*, generally between 0.5 and 5 MPa, with peak local stresses reaching up to 10 MPa (Brand, 2005). To better replicate these physiological conditions, the customized experimental setup developed in this work will allow to apply higher contact pressures than those achievable with conventional tribometers, by increasing the deformation applied to calibrated springs.

## 5 Conclusions

In conclusion, in this study PVA-based hydrogels to be used as artificial cartilage were developed mimicking the mechanical and tribological features of human articular cartilage. Among the tested concentrations, 20% PVA hydrogel demonstrated compressive stiffness within the physiological range and a relatively low friction coefficient, representing a suitable *in vitro* platform for evaluating joint lubricants. Friction tests confirmed that HA-based gels for viscosupplementation reduce the friction and wear of the PVA hydrogels, supporting their role in mimicking the protective function of synovial fluid *in vivo*. These findings support the possible use of 20% PVA hydrogels as reproducible artificial cartilage for preclinical assessment of viscosupplementation therapies.

## Data Availability

The original contributions presented in the study are included in the article/Supplementary Material, further inquiries can be directed to the corresponding author.
