# Supplementary material for: Development and tribological characterization of polyvinyl alcohol-based artificial cartilage for in vitro testing of viscosupplementation gels
Source: Front Bioeng Biotechnol. 2026 Jun 11;14:1770162. doi: 10.3389/fbioe.2026.1770162 (PMC13294369; doi:10.3389/fbioe.2026.1770162)
Supplement: Supplementary file 1 [file Supplementaryfile1.docx]

Supplementary Material

**1 Supplementary results on PVA frictional behavior**

Friction tests shown in Section 3.3 are limited to PVA hydrogel at 20% concentration. For the sake of completeness, results for PVA hydrogels at 15% and 25% concentration are here reported.

In **Supplementary Figure 1** a frequency-dependent variation in the friction coefficient is detected if using PBS as lubricant, with a higher friction coefficient at lower frequency. In the case of PVA hydrogels at 15% and 25% concentrations, the decrease in the friction coefficient is significantly different between low and high frequencies. Conversely, when HA-based gels are adopted as lubricants, no detectable frequency-dependent variation is observed.

For each testing frequency, the effect of the lubricant on PVA friction coefficient is evaluated. **Supplementary Figure 2** shows that, for all PVA concentrations tested, the use of HA-based gels as lubricant results in a decrease of the friction coefficient compared with PBS. Statistically significant differences between PBS and HA-based gels are detected only for some combinations. Additionally, HA lubrication reduces the variability in friction observed across different PVA concentrations, as shown in **Supplementary Table 1**.


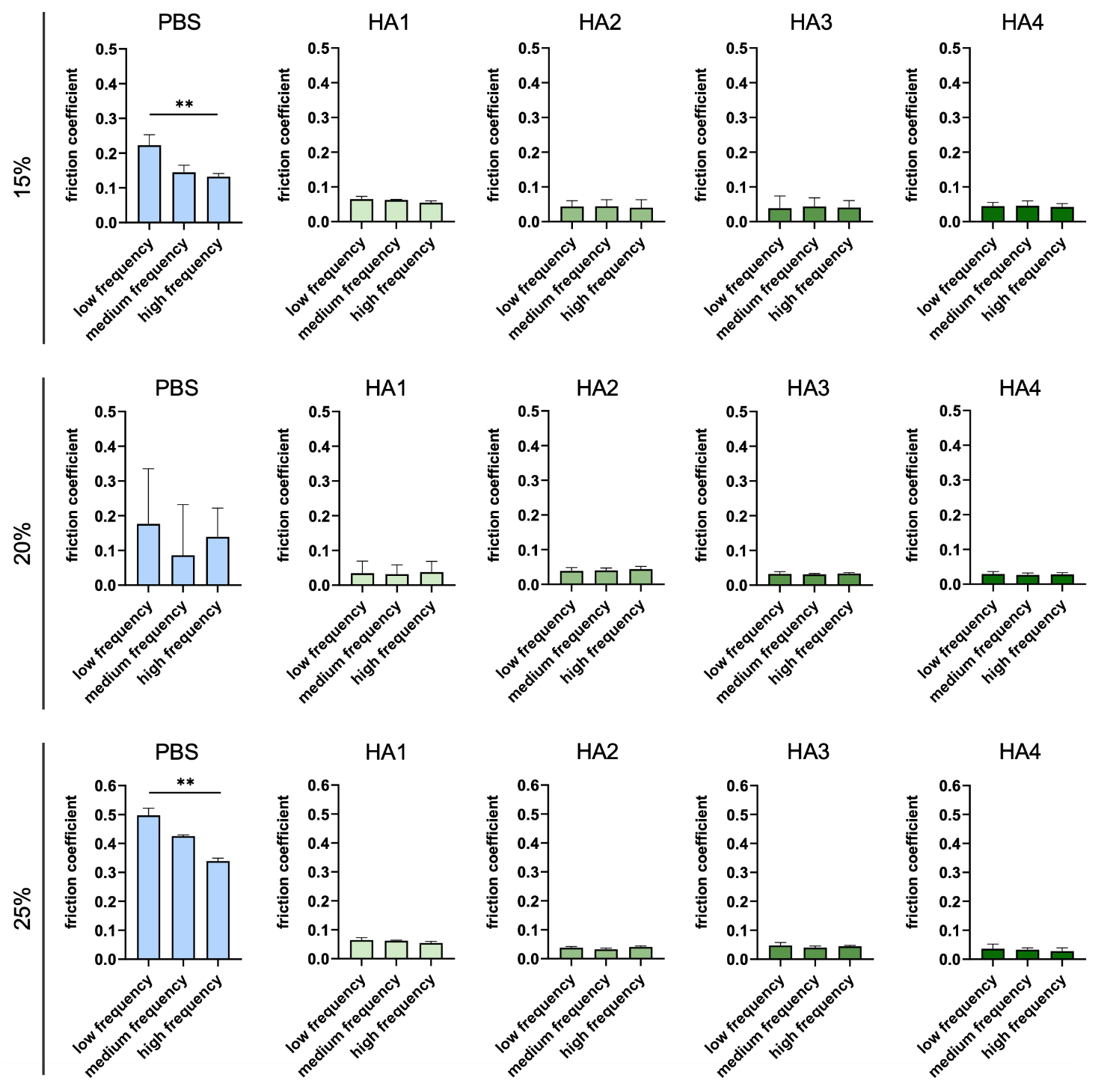


Supplementary Figure 1: Comparison of median friction coefficients ± IQR measured at three frequencies: low (0.05 Hz), medium (0.5 Hz), and high (2.5 Hz). Fifteen material–lubricant combinations are evaluated, i.e. PVA hydrogels at 15%, 20%, and 25% concentrations tested with PBS (A) or with HA-based gels (HA1, HA2, HA3, and HA4). **p < 0.01.


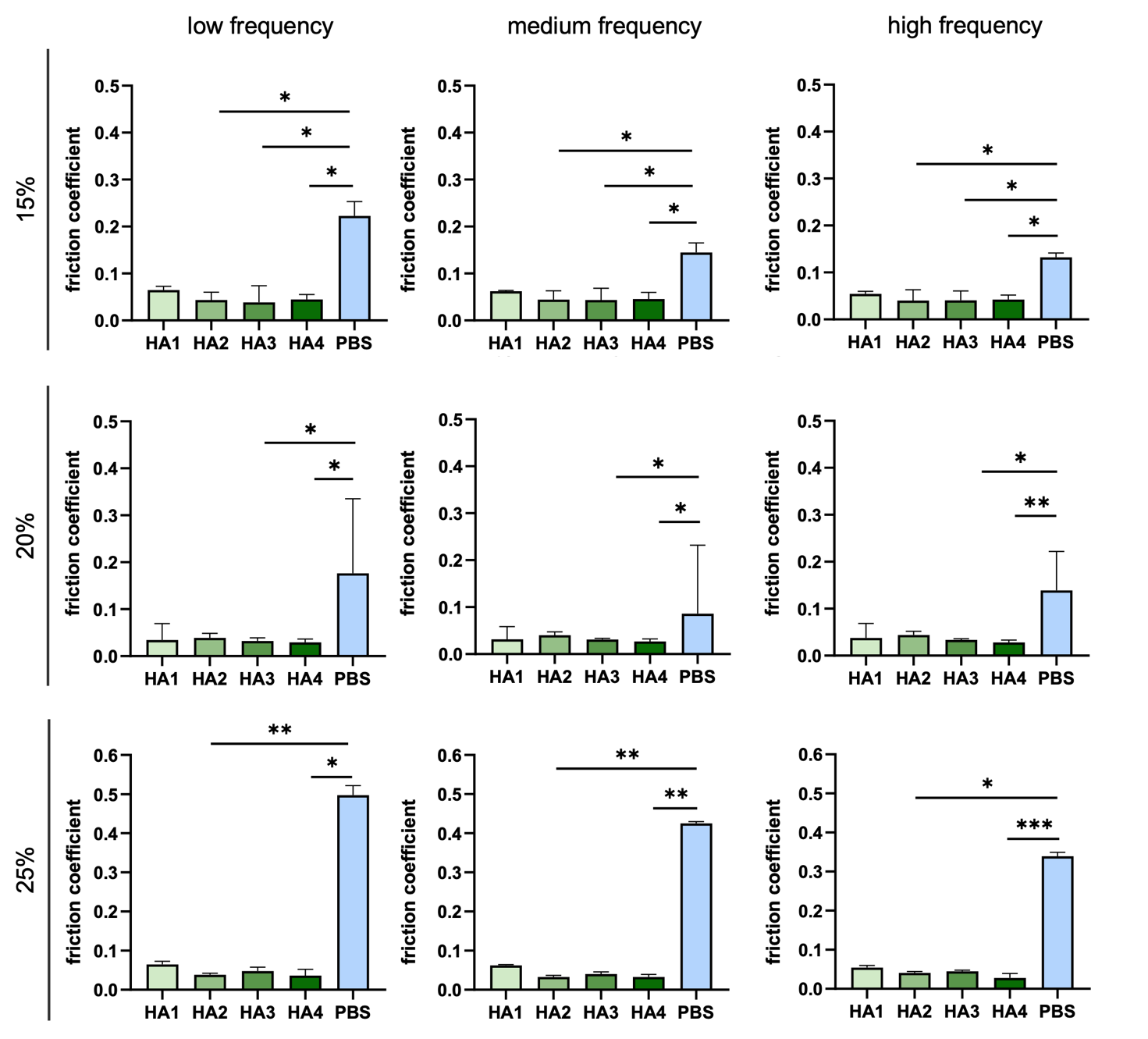


Supplementary Figure 2: Comparison of median friction coefficient ± IQR of PVA hydrogel at 15%, 20% and 25% concentration tested in different lubrification conditions, i.e. PBS or HA-based gels (HA1, HA2, HA3, HA4). Tests are carried out at low (0.05 Hz), medium (0.5 Hz), and high (2.5 Hz) frequencies. *p < 0.05, **p < 0.01, ***p < 0.001.


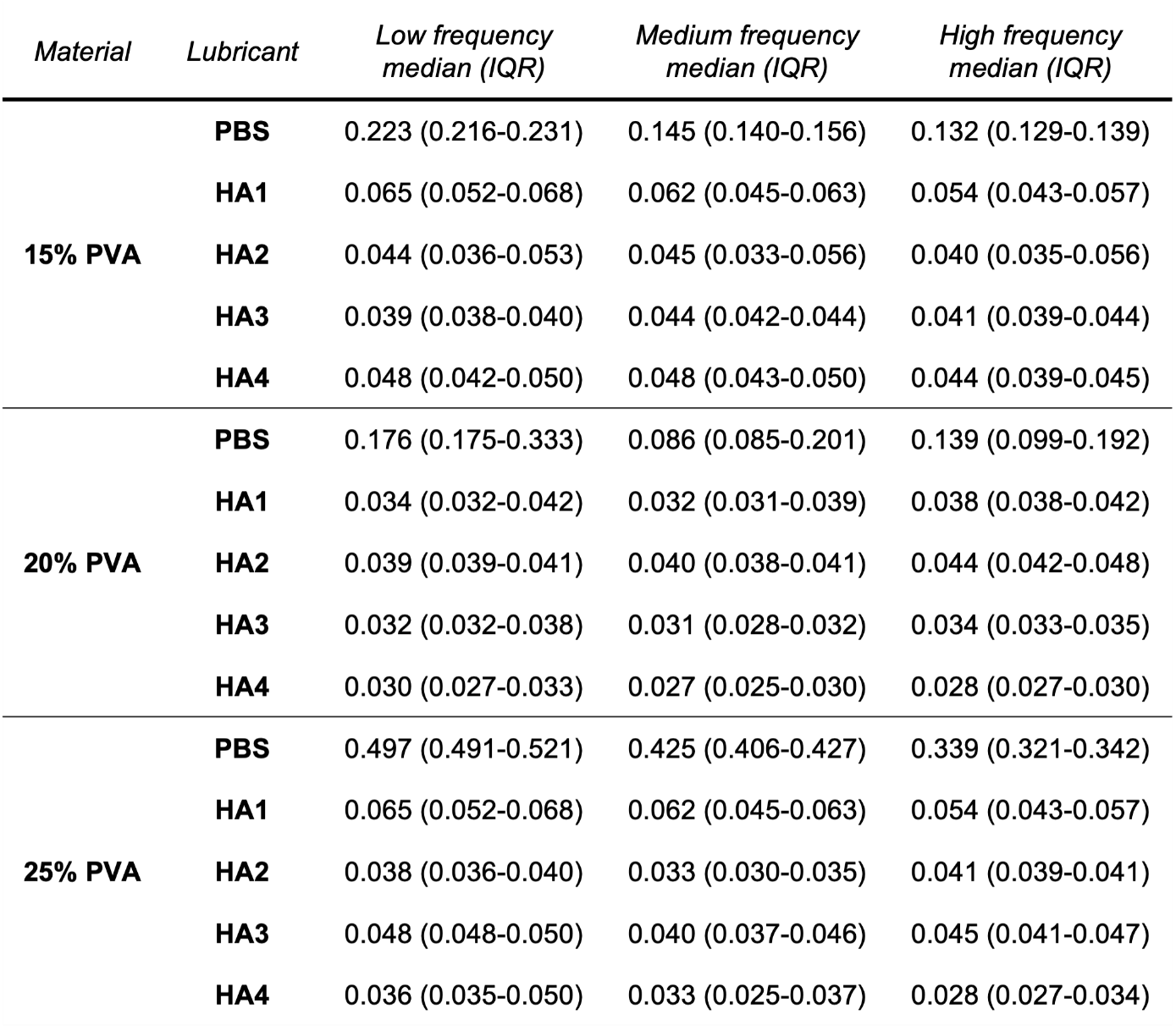


**Supplementary Table 1: Values of median friction coefficients, with corresponding IQR, measured at three frequencies: low (0.05 Hz), medium (0.5 Hz), and high (2.5 Hz). Values are reported for PVA hydrogels at 15%, 20%, and 25% concentrations tested with PBS or with HA-based gels (HA1, HA2, HA3, and HA4).**
